# Fibroblast Growth Factor 23 (FGF 23) and intact parathyroid hormone (iPTH) as markers of mineral bone disease among Nigerians with non-diabetic kidney disease

**DOI:** 10.4314/ahs.v22i1.42

**Published:** 2022-03

**Authors:** Yemi R Raji, Samuel O Ajayi, Abiodun M Adeoye, Olukemi Amodu, Bamidele O Tayo, Babatunde L Salako

**Affiliations:** 1 Department of Medicine, College of Medicine, University of Ibadan, Ibadan, Nigeria; 2 Department of Clinical Sciences, Nigerian Institute of Medical Research, Yaba, Lagos; 3 Institute of Child Health, College of Medicine, University of Ibadan, Ibadan, Nigeria; 4 Department of Public Health Sciences, Loyola University Chicago, Maywood, Illinois

**Keywords:** CKD, mineral bone disease, diagnostic, FGF 23, parathyroid hormone

## Abstract

**Background:**

Excess cardiovascular burden in patients with chronic kidney disease (CKD) has been attributed to the occurrence of CKD-Mineral Bone Disease (CKD – MBD). This study aimed to determine the spectrum of CKD-MBD among Nigerians with CKD using Fibroblast Growth Factor 23 (FGF 23) and intact Parathyroid Hormone (iPTH).

**Methods:**

Cross sectional survey of 105 patients with non-diabetic CKD and 104 controls. Information obtained were demographics, aetiology of CKD, features of CKD-MBD. Serum iPTH and FGF 23 were assayed.

**Results:**

The mean ages were 48.7±15.3 vs 48.6±17.4 years while 54.7% and 45.2% were males for cases and controls, respectively. The mean plasma FGF 23 (392.8±35.3 vs 133.8±22.7 RU/mL and plasma iPTH (289±25.6 vs 118±10.8 ng/L, respectively. The frequency of elevated FGF 23 (45.7% vs 24.0%, p<0.01) and abnormal iPTH (53.3% vs 14.1%, p- 0.01) were higher in cases. The prevalence of MBD were (59.0% vs 14.4%, p<0.01) in cases and controls while dialysis status OR 2.94, 95% CI (1.2803–5.3645), and elevated FGF 23 OR, 1.87, 95% CI (1.1782–5.4291) were associated with CKD-MBD.

**Conclusion:**

The study demonstrated high prevalence of CKD-MBD among patients with non-diabetic CKD while FGF23 and iPTH were useful assays in the diagnosis of CKD-MBD among Nigerians with CKD.

## Introduction

Chronic kidney disease (CKD) is a major contributor of disease burden among the sub-Saharan African population, it is associated with rising prevalence, poor awareness, excess cardiovascular disease (CVD) burden and high morbidity and mortality.^1^,^2^ The excess cardiovascular burden among CKD/end stage kidney disease (ESKD) has been well established and various mechanisms underpinning its occurrence have been described. Chronic Kidney Disease – Mineral Bone Disease (CKD – MBD) is a significant determinant of cardiovascular (CV) morbidity and mortality among this group of patients.^3^,^4^ CKD-MBD was defined by Kidney Disease Outcomes Quality Initiative (KDOQI in 2006 and Kidney Disease Improving Global Outcomes (KDIGO) in 2017 as either one or a combination of the following clinical situations: (a) abnormalities of calcium, phosphorus, parathyroid hormone (PTH), or vitamin D metabolism; (b) abnormalities in bone turnover, mineralization, volume, linear growth, or strength; (c) vascular or other soft tissue calcification.^5^,^6^ The occurrence of CKD-MBD starts early in the CKD and as early as stage II and progressively worsen as the kidney disease advances.^7^ Early manifestations are elevated serum phosphate and normal or low serum calcium. Studies have reported the prevalence of CKD-MBD among pre-dialysis patients to range between 30–50% and as high as 70% in patients with ESKD.^8^–^10^

The gold standard for assessment of CKD-MBD is bone biopsy and histology, which is invasive and not commonly used in routine clinical practice.^11^ The changes in biochemical parameters are surrogate markers of Mineral Bone Disease (MBD) that have been found to be useful in patients' clinical care. These biochemical parameters include serum calcium, phosphate, vitamin D and intact parathyroid hormone (iPTH).^11^,^12^ The biochemical parameters are used in conjunction with both the clinical information and bone dual X ray absorptiometry (DEXA).^13^ Recently, there has been a growing evidence supporting serum fibroblast growth factor 23 (FGF23) as a marker of CKD-MBD, in addition to being a correlate of cardiovascular disease (CVD).^14^ In normal homeostasis, FGF23 regulates and maintains a normal serum phosphorous. Serum FGF-23 is increasingly being adopted as a marker of CKD-MBD in routine clinical practice.^15^,^16^

In most of the sub-Saharan Africa, patients with CKD are either minimally or not evaluated for CKD-MBD at all, as majority of the patients pay out of pocket and thus add to the cost of care which is already not affordable by a large proportion of patients with CKD.^17^,^18^ Most centres in Nigeria therefore adopt preventive strategies with dietary phosphate restrictions, use of phosphate binders and vitamin D (Vit D).^19^ In addition, the indiscriminate use of phosphate binders and Vit D has been reported to be associated with low bone turn over (adynamic bone disease).^20^ This study aimed to determine the spectrum of CKD-MBD among patients with non – diabetic kidney disease in South West Nigeria.

## Materials and methods

### Study participants

A cross sectional study of non – diabetic kidney disease patients who were attending the Medical Outpatient Clinic of the University College Hospital, Ibadan and apparently healthy controls who were age and gender matched volunteers. Non-diabetic kidney disease was defined as participant with estimated Glomerular Filtration Rate (eGFR) less than 60mls/min/1.73m^2^ with or without albuminuria and who had no prior diagnosis of diabetes mellitus. Participants enrollment for the study was between 20^th^ August 2019 and 28^th^ February 2020. To be eligible participants must be 18 years and above, have diagnosis of CKD from non – diabetic kidney disease. Excluded from the study were individuals with Diabetes mellitus, kidney transplantation and those with history of parathyroidectomy.

### Informed consent and ethical approval

All participants gave written informed consent and ethical approval was obtained from the Joint University of Ibadan and University College Hospital institutional review board with the approval number UI/EC/19/0113.

### Clinical data

Relevant information was obtained using standard case report forms. All participants provided information on demographics, age of diagnosis, etiology and stage of CKD, symptoms and signs of CKD-MBD (bone pain, bone tenderness, bone swelling, pruritus, pathological fracture, generalized body weakness), history of co-morbidity, dialysis, dialysis vintage and medication history. Physical measurements obtained were weight, height and blood pressure.

### Laboratory measurements

All participants gave 20ml of blood, plasma intact parathyroid hormone (iPTH) was assayed in all participants using Enzyme Linked Immunosorbent Assay with ELISA kits (Cloud-Clone Corporation, United Kingdom). The manufacturer's range was 12.35–1,000pg/mL while the intra and inter-assay coefficients were <10% and < 12%, respectively. The plasma c-terminal FGF 23 (cFGF-23) was analyzed using ELISA assay kits (Cloud-Clone Corporation, United Kingdom). The manufacturer's range was 7.8 – 500RU/ml (15.6–1,000pg/mL) while the intra and inter-assay coefficients were <10% and < 12%, respectively. Both assays were analyzed using Emax ELISA Reader (Molecular Device, United States). Serum electrolytes, calcium and phosphate were determined using spectrophotometric method with a semi-autoanalyzer (Jenway Spectrophotometer 6305 series, United Kingdom). eGFR was estimated from the serum creatinine using Chronic Kidney Disease Epidemiology Collaboration (CKD-EPI) equation.

### Statistical analysis

Data obtained was entered into Microsoft Excel and was subsequently transferred to the Statistical Package for Social Science version 23 for analysis. Baseline descriptions of socio-demographic and clinical variables were reported as proportions for categorical variables and mean (standard deviation) for continuous variables. The association between categorical variables was tested using chi-square analysis while Student's T - test was used to test association between continuous variables. A logistic regression analysis was employed to determine factors independently associated with CKD-MBD. Statistical significance was set as p-value <0.05.

### Operational definitions

Elevated cFGF 23 was defined as FGF 23 above 104RU/ml 21 while elevated iPTH was defined as iPTH above 65ng/L.22 Hypocalcaemia was defined as serum calcium < 2.1mMol/L23 and hypercalcaemia was defined as serum calcium > 2.5mMol/L.24. Hyperphosphatemia was defined as serum phosphate > 1.45mMol/L.25 High turn over mineral bone disease was defined as elevated iPTH > 65ng/L while low turn over mineral bone disease was defined as iPTH < 10ng/L.26 CKD-MBD was defined as abnormality in one or more of serum iPTH, calcium and phosphate.^27^

## Results

A total of 209 participants were enrolled for the study, 105 were individuals with non-diabetic kidney disease while 104 were healthy controls. The mean ages for cases and controls were 48.7±15.3 vs 48.6±17.4 years, respectively. The males accounted for 54.7% of cases and 45.2% of controls, Table 1. The mean body mass index (BMI) was significantly lower among the cases compared to the controls (23.5±3.8 vs 26.3±5.6kg/m^2^) while the mean DBP (84.1 vs 79.8 12.3mmHg) and SBP (140.1±22.8 vs 130.1±19.6mmHg) were higher among cases, Table 1. More than two-third of both the cases and controls had at least a secondary school education, and hypertension and chronic glomerulonephritis (CGN) were the two leading causes of CKD among the cases, Table 1.

**Table 1:** Baseline characteristics of participants

Variable	Non-Diabetic CKD n = 105 Mean (SD)/frequency (%)/Median/ (interquartile range)	Healthy Controls n = 104 Mean (SD)/frequency (%) /Median/ (interquartile range)
Age (years)	48.7±15.3	48.6±17.4
Gender Male Female	57 (54.3%) 48 (45.7%)	47 (45.2%) 57 (54.7%)
BMI (kg/m^2^)	23.5±3.8	26.3±5.6
DBP (mmHg)	84.1 15.9	79.8 12.3
SBP (mmHg)	140.1±22.8	130.1±19.6
Educational Status No formal education Primary education Secondary education Tertiary education	6(5.7%) 21(20.0%) 27(25.7%) 51(48.6%)	9(8.6%) 19(18.3%) 25(24.1%) 51(49.0%)
Aetiology of CKD Hypertension CGN SCN Others	42 (40.0%) 35 (33.3%) 16 (15.2%) 12 (11.5%)	
Stages of CKD Stage III Stage IV Stage V	44 (41.9%) 24 (22.9%) 37 (35.2%)	
Dialysis status Non-dialysis Dialysis	64 (61.0%) 41(39.0%)	
Dietary phosphate restriction	43 (41.0%)	13 (12.5%)
Medications Phosphate binders Vitamin D supplement*	75 (71.4%) 56 (53.3%)	7 (6.7%) 14 (13.5%)
Serum Creatinine (µMol/L)	632 ± 34.2	106 ± 15.8
Serum Calcium (mMol/L)	1.9±0.8	2.2±1.2
Serum Phosphate (mMol/L)	1.48 (0.16 -5.39)	1.18 (0.82 – 5.29)
Serum Albumin (g/dL)	5.1±2.1	6.0±1.7
Calcium Phosphate product	12.10 (4.85 – 74.39)	7.81 (4.10 – 50.01)
Plasma cFGF2 (RU/mL)	392.8 ± 35.3	133.8 ± 22.7
Plasma iPTH (ng/L)	289 ± 25.6	118 ± 10.8
Urinary Albumin Creatinine ratio (mg/g)	178.12 (7.89 – 1,050.0)	25.32 (0.65 – 123)

BMI – Body Mass Index, CGN – Chronic Glomerulonephritis, CKD – Chronic Kidney Disease, DBP – Diastolic Blood Pressure, FGF 23 – Fibroblast Growth Factor 23, iPTH – Intact Parathyroid Hormone SBP – Systolic Blood Pressure, SCN – Sickle Cell Nephropathy, SD -Standard Deviation

The mean serum creatinine (632 ± 34.2 vs 106 ± 15.8µmol/L)), plama FGF23 (392.8 ± 35.3 vs 133.8 ± 22.7 RU/mL), plasma iPTH (289 ± 25.6 vs 118 ± 10.8 ng/L), while the median urinary albumin creatinine ratio (UACR) 178.12 (7.89 – 1,050.0) vs 25.32 (0.65 – 123) mg/g and median calcium phosphate products 12.10 (4.85 – 74.39) vs 7.81 (4.10 – 50.01)mMol/L were higher among the cases compared to the controls, Table 1.

**Figure 1:**
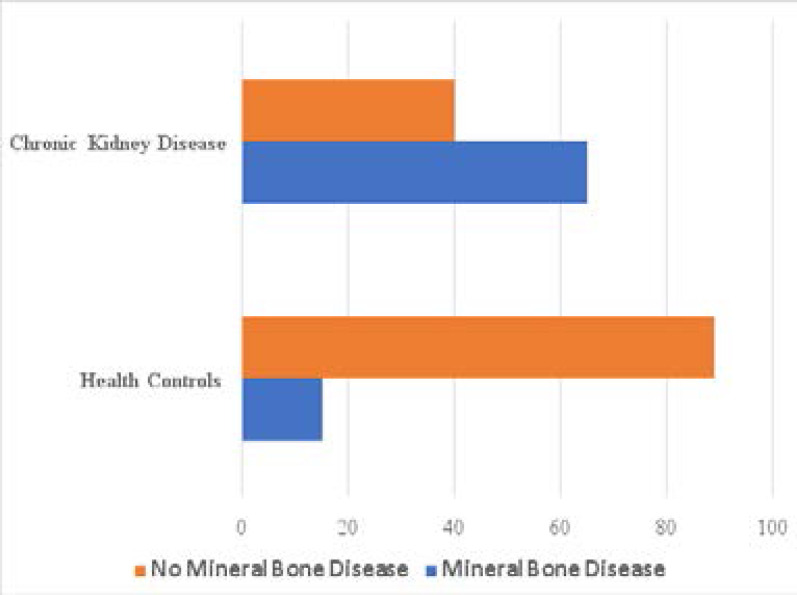
Proportions of participants with Chronic Kidney Disease and health controls with Mineral Bone Disease

The frequency of Hypocalcemia (41.3%vs 17.3%, p<0.01), hyperphosphatemia (48.6% vs 13.3%, p -0.01), elevated FGF 23 (45.7% vs 24.0%, p<0.01), abnormal iPTH (53.3% vs 14.1%, p -0.01) were significantly higher among the cases compared to the controls, Table 2. The proportions of participants with Mineral Bone Disease (MBD) was significantly higher in the cases compared to the controls (59.0% vs 14.4%, p<0.01), Figure 1.

**Table 2:** Pattern of mineral bones disease among participants with non – diabetic CKD and controls

Variable	Non-Diabetic CKD n = 105 frequency (%)	Healthy Controls n = 104 frequency (%)	95%CI	P - value
Hypocalcemia	43 (41.3%)	18 (17.3%)	3.31 (1.7475–6.2832)	< 0.01
Hypercalcemia	19 (18.1%)	11 (10.6%)	1.87 (0.8487 – 4.1503)	0.21
Hyperphosphatemia	51 (48.6%)	19 ((18.3%)	4.23 (2.2557 – 7.9140)	0.01
Elevated plasma FGF 23	48 (45.7%)	25 (24.0%)	2.66 (1.4721 – 4.8071)	< 0.01
Elevated plasma iPTH	39 (37.1%)	8 (7.7%)	7.10 (3.1145 – 16.1443)	< 0.01
Reduced plasma iPTH	17 (16.2%)	7 (6.7%)	2.68 (1.0601 – 6.7597)	0.03
Abnormal iPTH	56 (53.3%)	15 (14.4%)	6.78 (3.4767 – 13.2256)	0.01
Overall Mineral Bone Disease	62 (59.0%)	15 (14.4%)	8.56 (4.7322 – 16.7392)	< 0.01
**Types of Mineral Bone Disease** High bone turn over MBD Low turn bone turn over MBD Unclassified	39 (37.1%) 17 (16.2%) 6 (5.7%)	8 (7.7%) 7 (6.7%) 0 (0%)	7.20 (3.4538 – 14.6522)	0.01

dialysis status Odd Ratio (OR), 2.94 95% Confidence Interval (CI) (1.2803 – 5.3645) and elevated FGF 23 OR 1.87, 95% CI (1.1782 – 5.4291) were independently associated with CKD-MBD, Tables 3.

The symptoms of CKD-MBD were observed to be significantly higher among the cases than controls bone pain (26.7 vs 8.7, p<0.01), pruritus (23.8% vs 5.7%, p<0.01), skin rash (21.9% vs 11.5%, p-0.04) and myalgia (27.6% vs 10.7%, p<0.01), Table 4

**Table 3:** Factors associated with chronic kidney disease – mineral bone disease

Variables	Unadjusted Odd Ratio (95% Confidence Interval)	p-value	**Adjusted Odd Ratio (95% Confidence Interval)	p-value
Educational status (Tertiary)	0.72 (0.3281 – 1.5631)	0.40	0.79 (0.8135 – 2.5392)	0.17
Ethnicity (Yoruba)	1.68 (0.7138 – 3.9472)	0.23	1.61 (0.6734 – 4.9376)	0.44
BMI > 25kg/m^2^	0.95 (0.4217 – 2.1676)	0.91	1.03 (0.6153 – 3.7115	0.72
Stage of CKD	1.03 (0.4545 –-3.3192	0.95	0.75 (0.3917 – 3.7830	0.64
Elevated cFGF23	2.99 (1.3597 – 6.8054)	< 0.01	1.87 (1.1782 – 5.4291)	< 0.01
Dialysis status (ESRD)	3.41 (1.4338 – 8.1258)	0.02	2.94 (1.2803 – 5.3645)	0.02
Dietary phosphate restriction	0.95 (0.4339 – 2.0672)	0.89	0.82 (0.3862 – 6.2563	0.25
Treatment for CKD MBD	0.80 (0.3637 – 1.7599)	0.58	1.07 (0.7527 – 2.9461)	0.75
*Co-morbidities	0.73 (0.3304 – 1.5965)	0.43	0.89 (0.2590 – 1.3042)	0.21

BMI – Body Mass Index, CKD – Chronic Kidney Disease, CKD-MBD – Chronic Kidney Disease Mineral Bone Disease, ESRD – End Stage Renal Disease, FGF 23 – Fibroblast Growth Factor 23.

Comorbidities: The co-morbidities include heart failure, hypertensive heart disease, obesity and athritis.

Adjusted for age, gender and aetiology of chronic kidney disease.

**Table 4:** Clinical features of chronic kidney disease – mineral bone disease among participants CKD – Chronic Kidney Disease

Variable	Non-Diabetic CKD n = 105 Mean (SD)/frequency (%)	Healthy Controls n = 104 Mean (SD)/frequency (%)	p - value
Bone pain	28 (26.7%)	9 (8.7%)	<0.01
Bone deformity	2 (2.0%)	0 (0%)	0.16
Previous fracture	7 (6.7%)	3 (2.9%)	0.20
Skin ulcers	2 (2.0%)	0 (0%)	0.16
Skin rash	23 (21.9%)	12 (11.5%)	0.04
Pruritus	25 (23.8%)	6 (5.8%)	<0.01
Myalgia	29 (27.6%)	11 (10.7%)	<0.01
Muscle weakness	6 (5.7%)	2 (1.9%)	0.15
Tendon rupture	8 (7.6%)	3 (2.7%)	0.13

## Discussion

CKD-MBD has been underdiagnosed among patients with CKD in sub-Saharan because the tests required for the diagnosis of CKD-MBD are not readily available and where available were not affordable by majority of the patients, who often pay out of pockets. And such, less attention is sometimes paid to the clinical features of CKD-MBD unlike that of the symptoms of uraemia and anaemia which are commonly looked for during the routine medical consultations for individuals with CKD. Majority of the participants with the non-diabetic CKD were caused by hypertension andCGN and more than 60% were pre-dialysis CKD.

The average iPTH hormone was higher among patients with CKD compared to the healthy controls in addition to the high prevalence of elevated iPTH (59.0%) in the cohort with CKD. This finding is similar to the report by Abdu et al 19, Okoye et al 20, Chuang et al 28 and Ahmed et al 29 who reported 58.0%, 80.0%, 54.7% and 55.0%, respectively. The prevalence in all these studies were based on KDIGO definition of CKD-MBD. Majority of the participants with CKD-MBD in this study had features suggestive of high turn over mineral bone disease (MBD) 60% while 27.4% had features of low turn over MBD. The pattern observed is similar to earlier reports among predialysis and ESKD patients with CKD-MBD.^28^,^29^

The prevalence of CKD-MBD varies with different markers of CKD-MBD, abnormal plasma iPTH (45.7%) and elevated plasma FGF-23 (53.3%). The abnormal iPTH represents elevated and low level of iPTH and are commonly seen among patients with CKD and has a direct correlation with severity of CKD. Suprisingly, a relatively high proportion (14.4%) of the controls had mineral bone disease (BMD) that were not related to CKD, the finding buttresses the fact that other causes of MBD may be prevalent among the controls. The secondary causes of MBD reported to be common in the population studied include osteoporosis, indiscriminate use of vitamin D analogues and calcium based medications.^30^–^33^ Exclusion of individuals with Diabetes mellitus may be responsible for the low prevalence of low turn over MBD in this study. Factors that increase the risk of low turn over CKM-MBD include Diabetes mellitus, the use of vitamin D analogues and phosphate binders.^34^ The vitamin D analogues are often prescribed to patients with CKD, usually without assay of iPTH and FGF 23, the two assays are not readily available in low income settings and where available are often out of reach for most patients with CKD.

Plasma FGF 23 as a marker of CKD-MBD rises early in the disease compared to iPTH, and could be elevated as early as stage 2 CKD. FGF 23 is a phosphaturia hormone and are elevated in diseases associated with increased phosphate production or decrease phosphate excretion.^35^ In addition, FGF 23 is a marker of CVD among individuals with CKD. The use of serum FGF 23 in addition to iPTH have been reported to enhance early detection of CKD-MBD and increases the predictive ability of cardiovascular events in patients with CKD.^36^ Furthermore, in a prospective study of 227 African American and 1633 non-African American participants with nondiabetic CKD, increased FGF 23 levels was associated with significantly increased risk for progressive CKD and this effect was independent of the mostly normal serum phosphate levels.^37^ In incident ESRD patients on haemodialysis increased FGF 23 levels associated independently with increased risk for mortality.^38^ We observed a normally distributed pattern of plasma FGF 23 and intact iPTH in the patients with CKD, which is at variance with reports from study in similapopulation.^39^ The widespread use of phosphate binders and vitamin D analogues among this cohort could have been responsible for the observed difference. 32,33

Although, FGF 23 has been reported to be homogenous in most population, its level is lower by 20 – 30% among the African Americans and Hispanics compared to the Caucasians.^40^ Similarly, a study among South African population observed lower plasma FGF 23 among the Africans compared to the Caucasian population.^39^ The plasma FGF 23 cut off of 104RU/ml used in this study was based on the plasma level among the African Americans. 21 Furthermore, the observed mean plasma FGF 23 was also high in the control group (133.8 ± 22.7 RU/ml), this could be explained by the finding of about 15% of the controls with MBD, coupled with the fact that the exact reference range of plasma FGF 23 level in the studied population was not known.

Hyperphosphatemia was observed in 48.6% of patients with CKD, this is similar to reports from Abdu et al 19 and Okoye et al 20 who reported the prevalence of hyperphosphatemia in similar West African populations to be 39.5% and 69.4%, respectively. Hyperphosphatemia is an early finding in CKD-MBD and it is usually prevented through dietary phosphate restrictions, the use of phosphate binders, and use of dialysis in patients with ESKD. Hypocalcemia was 41.3% among patients with non-diabetic kidney disease and similar to 46% reported by Gimba et al 41.

The leading symptoms of CKD-MBD observed among patients with CKD were myalgia (27.6%), bone pain (26.7%), pruritus (23.8%) and skin rash (21.9%). These symptoms are non-specific and could be explained by other features of CKD that include uraemia and anaemia. These symptoms are similar in both high and low output CKD-MBD spectrums and it is pertinent to mention that the absence of symptoms as observed in about half of the patients with CKD-MBD does not exclude the disease.

The study is not without its limitations, since patients with Diabetic kidney disease were excluded from the study, the findings of this study can be generalised to every individual with CKD. The relatively small sample size also makes it difficult to generalise the findings of this study. However, the study made use both plasma FGF 23 and iPTH in making diagnosis of CKD-MBD.

## Conclusion

The plasma FGF 23 and iPTH were useful assays in the diagnosis of CKD-MBD among Nigerians with CKD.

## Data Availability

The data used to support the findings of this study are available from the corresponding author upon request.
